# The Virulence Polysaccharide of *Salmonella* Typhi Suppresses Activation of Rho Family GTPases to Limit Inflammatory Responses From Epithelial Cells

**DOI:** 10.3389/fcimb.2019.00141

**Published:** 2019-05-08

**Authors:** Farhat Parween, Jitender Yadav, Ayub Qadri

**Affiliations:** Hybridoma Laboratory, National Institute of Immunology, New Delhi, India

**Keywords:** *Salmonella* Typhi, Vi polysaccharide, prohibitin, Cdc42, Rac1

## Abstract

Vi capsular polysaccharide (Vi) is a major virulence factor of human typhoid-causing pathogen *Salmonella enterica* serovar Typhi (*S*. Typhi). It distinguishes *S*. Typhi from closely related non-typhoidal *Salmonella* serovars such as *S*. Typhimurium which do not normally cause systemic infection in humans. Vi not only forms a capsule around *S*. Typhi but it is also readily released from this pathogen. We have previously reported that Vi targets prohibitin to inhibit cellular responses activated through immune receptors. Here, we show that engagement of membrane prohibitin with Vi prevents *Salmonella*-induced activation of small Rho-family GTPases, Rac1, and Cdc42, and suppresses actin cytoskeletal rearrangements resulting in reduced invasion and highly subdued inflammatory responses. Cells infected with *S*. Typhimurium in the presence of Vi show poor activation of NF-kB and MAP-kinase pathways of intracellular signaling. Treatment with Vi brings about redistribution of Rac-1, prohibitin, and ganglioside GM1 in membrane raft domains. Vi-mediated interference with activation of Rho-family GTPases represents a previously unrecognized mechanism by which *S*. Typhi can limit its invasion and alarming of the host.

## Introduction

Pathogenic serovars of bacterium *Salmonella enterica* such as *S*. Typhi and *S*. Typhimurium infect their hosts by invasion of non-phagocytic intestinal cells. This invasion is mediated by intracellularly delivered *Salmonella* effectors including SopE, SipA, and SipC that are encoded by genes on a specialized region of *Salmonella* chromosome called *Salmonella* Pathogenicity Island 1 (Srikanth et al., 2011). These effectors cause the activation of membrane GTPases and bring about actin cytoskeletal rearrangements resulting in membrane ruffling and bacterial uptake into the epithelial cells (Hardt et al., 1998). This activation also stimulates the intracellular signaling pathways mediated by NF-kB and MAP-kinases which in turn trigger nuclear responses that lead to induction of inflammatory cytokines and chemokines (Bruno et al., 2009). The clinical outcome of infection with *Salmonella* depends on the serovar and the type of host. *Salmonella* Typhi causes systemic infection, typhoid, in humans while non-typhoidal serovars such as *S*. Typhimurium produce only localized gastroenteritis (Young et al., 2002; Dougan and Baker, 2014). Typhoid continues to be a major public health problem with more than 20 million cases and 200,000 deaths every year world wide (Dougan and Baker, 2014). The reasons for different clinical manifestations produced by *S*. Typhi and *S*. Typhimurium are not completely understood. The two are very closely related and share close to 90% homology at the genome and the proteome level (Napolitani et al., 2018). The functions of serovar-specific proteins have not been worked out. *S*. Typhimurium is believed to generate a potent inflammatory response that brings about its clearance in the human gut. On the other hand, *S*. Typhi proceeds stealthily and disseminates systemically by manipulating gut-generated innate immune responses (Santos, 2014). Although the mechanisms by which *S*. Typhi brings about these manipulations have not been completely elucidated, several studies have suggested that virulence factors encoded by genes of other *Salmonella* Pathogenicity Islands might be implicated (Vernikos and Parkhill, 2006; Schadich et al., 2016).

A major distinction between *S*. Typhi and *S*. Typhimurium is the presence of virulence capsular polysaccharide, Vi, in *S*. Typhi. Vi is a linear polymer of α1,4 2-N-acetyl galacturonic acid with variable O-acetylation at C-3 position. Its biosynthesis and export is associated with genes of viaB locus located in *Salmonella* Pathogenicity Island 7, a serotype specific cluster not present in all *Salmonella* serovars (Pickard et al., 2003; Seth-Smith, 2008; Schadich et al., 2016). Vi encapsulates *S*. Typhi and it is also released from this bacterium during its *in vitro* growth as well as during its interaction with host cells (Santhanam et al., 2014). We and others have previously shown that Vi can enable *S*. Typhi to evade TLR-driven host defense responses from intestinal epithelial cells (IECs) and monocytes (Sharma and Qadri, 2004; Raffatellu et al., 2005; Garg and Qadri, 2010). Our studies with these cell types and T cells showed that Vi suppresses immune responses by directly targeting membrane associated prohibitin family of molecules, which are highly conserved ubiquitously expressed proteins involved in cell signaling, regulation of mitochondrial respiratory chain complexes, and gene expression (Sharma and Qadri, 2004; Garg and Qadri, 2010; Santhanam et al., 2014; Peng et al., 2015). In contrast, studies by Baumler and his group suggested that the expression of Vi might inhibit inflammatory and innate immune responses mainly by hampering the release of bacterial effectors that are critical for initiating these responses (Wilson et al., 2008; Winter et al., 2008). Here, we present evidence that engagement of membrane prohibitin with Vi inhibits *Salmonella*-induced activation of GTPases, Rac1, and Cdc42, thereby reducing bacterial intake and suppressing secretion of cytokines.

## Results

### Vi Inhibits Invasion of *Salmonella* Into Epithelial Cells

**Figure 1:**
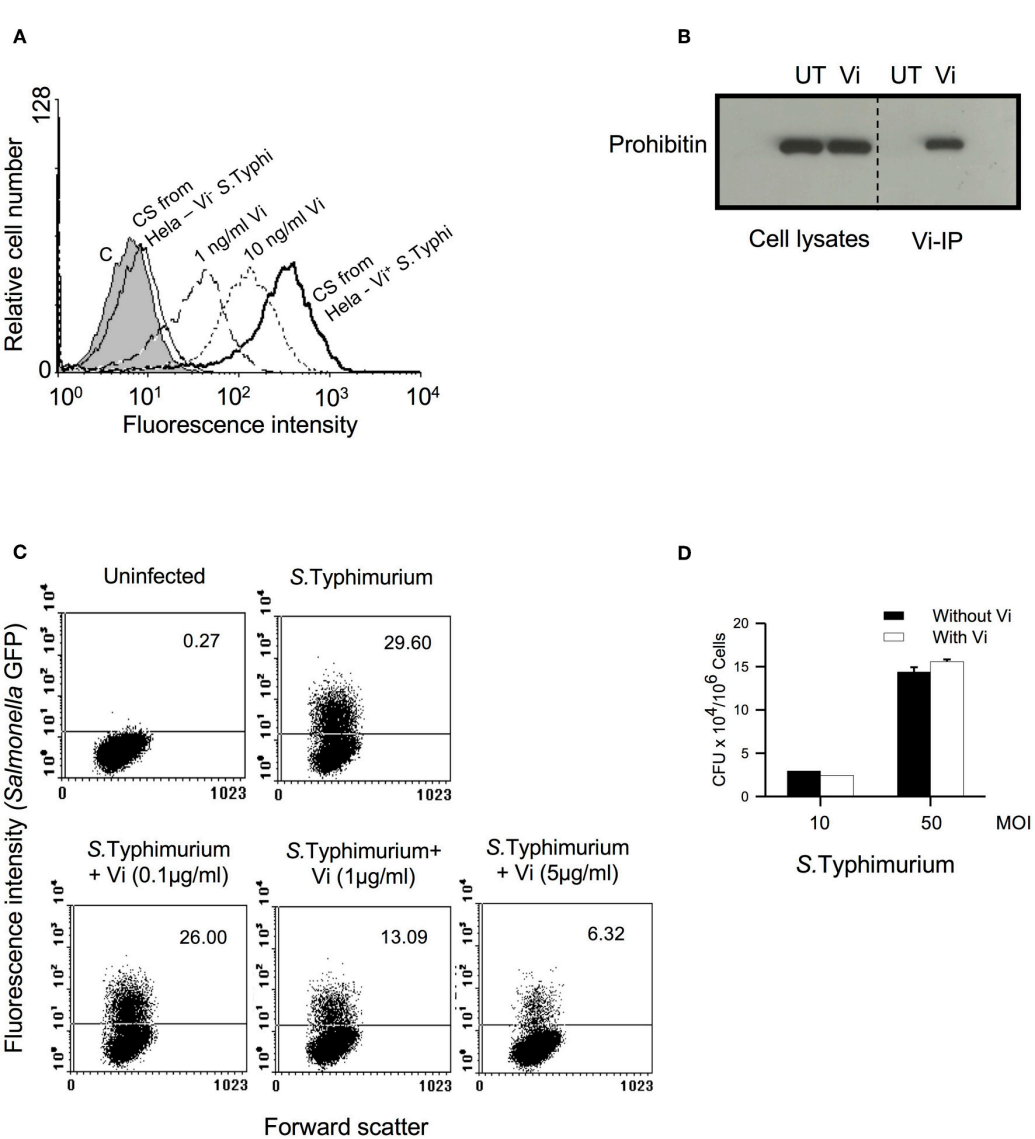
Vi interacts with membrane prohibitin and inhibits invasion of epithelial cells with *Salmonella*. **(A)** Hela cells were incubated for 1 h at 4°C with different concentrations of Vi or cell-free culture supernatants (C.S) obtained from co-cultures of Hela cells with Vi^+^ or Vi^−^ *S*. Typhi (100 MOI). Cells were washed and incubated with anti-Vi MoAb followed by FITC-labeled anti-mouse Ig Ab. Shaded histogram represents control cells, which were incubated only with anti-Vi MoAb and FITC-anti-mouse Ig Ab. Cells were analyzed by flow cytometry. CS from Hela-*S*. Typhi-culture supernatant from Hela-*S*. Typhi co-culture. **(B)** Cells were incubated with Vi at 4°C for 1 h and lysed with Triton X-100 (1%). Vi-interacting proteins were immunoprecipitated from cell lysates with anti-Vi MoAb loaded on Protein G-Sepharose. Proteins were electrophoresed in a 12% SDS-polyacrylamide gel, transferred to nitrocellulose membrane and probed with anti-prohibitin antibody. The blot was developed using ECL. UT, untreated cells; Vi, Vi-treated cells. **(C)** Cells were infected with GFP^+^ *S*. Typhimurium (50 MOI) in the absence or presence of indicated concentrations of Vi, washed to remove unbound bacteria and incubated for another 30 min in presence of gentamycin (100 μg/ml). Cells were fixed with paraformaldehyde and analyzed by flow cytometry. **(D)** Cells were incubated with *S*. Typhimurium in the absence or presence of Vi at 4°C, washed to remove unbound bacteria and lysed with PBS containing 0.1% Triton X-100. CFUs were determined by plating cell lysates on SS-Agar. Data are representative of 2 independent experiments.

Our previous study with T cells had shown that engagement of membrane prohibitin with Vi brings about actin depolymerisation in these cells and suppresses TCR-activated cellular responses (Santhanam et al., 2014). Since *Salmonella* invasion is mediated through induction of actin cytoskeletal rearrangements, we reasoned that interaction of Vi with prohibitin might also modulate the ability of epithelial cells to enable bacterial invasion. We tested this possibility using Hela-*S*. Typhimurium culture system which has been extensively used to study invasion of cells with pathogenic *Salmonella* (Malik-Kale et al., 2011). Consistent with our previous studies, Vi interacted with Hela cells in a dose-dependent manner and it specifically recognized membrane associated prohibitin from these cells (Figures 1A,B). This interaction was also seen with Vi released during co-culture of Vi positive *S*. Typhi with cells (Figure 1A). Strikingly, Vi-binding inhibited invasion of Hela cells with *S*. Typhimurium in a dose-dependent manner (Figure 1C). Similar inhibition was seen upon infection of human colonic epithelial cell line T84 with *S*. Typhimurium (Supplementary Figure S1). Vi did not reduce adherence of *S*. Typhimurium to cells which suggested that Vi-prohibitin interaction might be inhibiting bacterial intake by modulating pathogen-initiated signaling involved in invasion (Figure 1D).

### Engagement With Vi Suppresses Activation of Rho Family GTPases and Induction of Inflammatory Responses in Cells Infected With *Salmonella*

**Figure 2:**
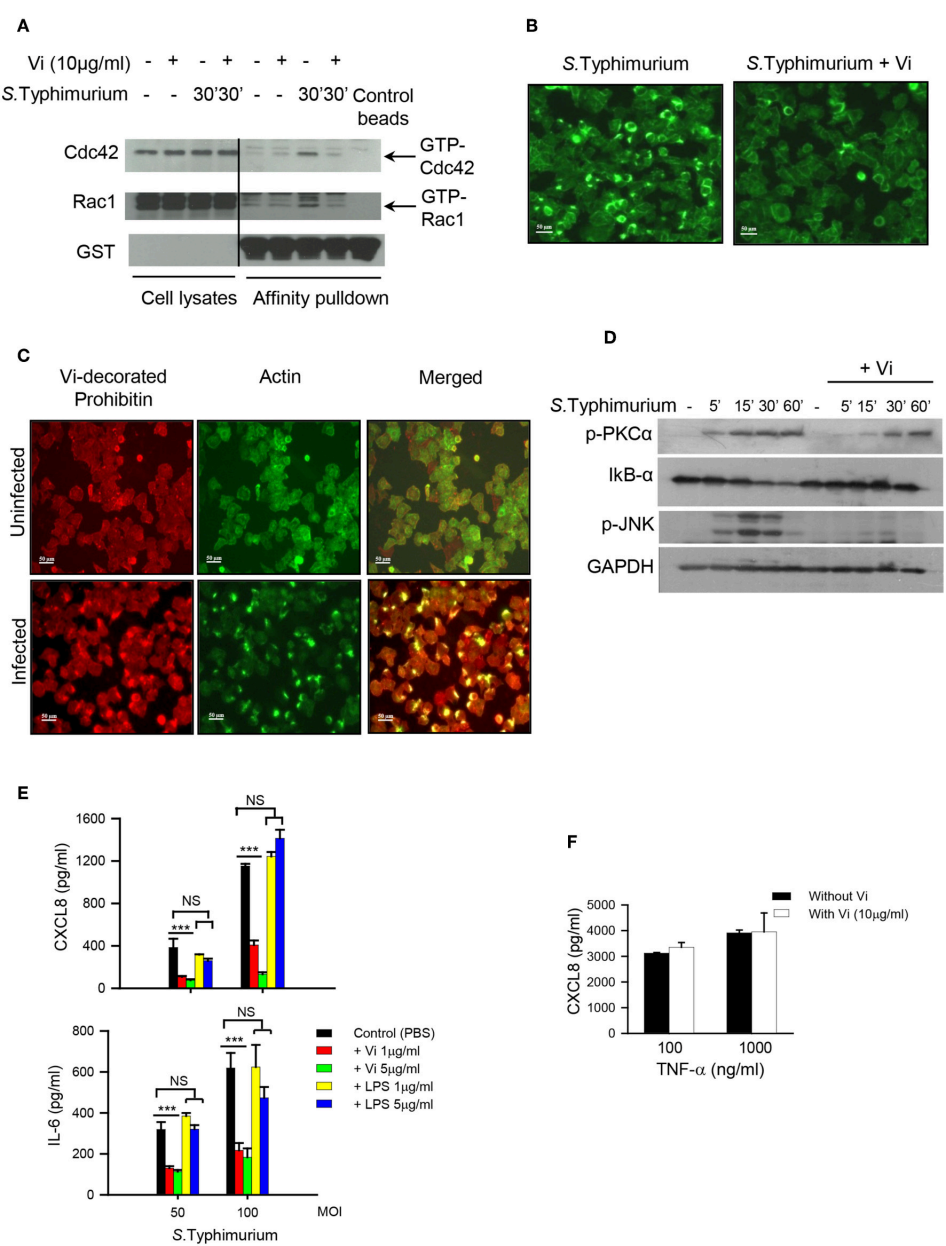
Vi suppresses activation of Cdc42 and Rac-1 in *Salmonella* infected cells. **(A)** Hela cells were infected with *S*. Typhimurium in the absence or presence of Vi and lysed with Triton X-100 (1%). Active Cdc42 and Rac1 (GTP-bound form) were precipitated from cell lysates by affinity pull-down assay using Cdc42/Rac1-binding domain of p21 activated kinase-1 (PAK). PAK-bound proteins and cell lysates were electrophoresed in 10% SDS-polyacrylamide gel and after transferring proteins to a nitrocellulose sheet, immunoblotted with specific antibodies to Cdc42, Rac1, and GST. The blot was developed with ECL. **(B)** Untreated or Vi-treated (30 min at 37°C) cells were infected with *S*. Typhimurium (100 MOI) for 30 min. Cells were fixed with paraformaldehyde and incubated with Alexa-fluor 488-labeled Phalloidin to stain filamentous actin. Cells were visualized under a fluorescence microscope (Nikon TE2000) and images were imported into Adobe Photoshop. **(C)** Uninfected or *S*. Typhimurium - infected (100 MOI for 30 min) cells were stained for filamentous actin with Alexafluor-488-labeled Phalloidin (green staining). Subsequently, cells were incubated with Vi at 4°C (for prohibitin localization) followed by anti-Vi antibody and Alexafluor 594-labeled anti-mouse Ig Ab (red staining). Images were merged in Adobe Photoshop to see co-localization of F-actin with Vi-bound prohibitin. **(D)** Cells were treated with Vi or PBS and infected with *S*. Typhimurium at 100 MOI. The expression of IkB-α, p-JNK and p-PKC-α was evaluated at different time points by western blotting using specific antibodies. The blot was subsequently probed with anti-GAPDH antibody and developed using ECL. **(E)** Cells (in triplicate) were treated with indicated concentrations of Vi, LPS or equivalent volume of PBS for 30 min before infecting with *S*. Typhimurium at different MOIs for 30 min. Cells were washed and incubated for another 6 h in serum-free RPMI-1640 supplemented with gentamycin. CXCL8 and IL-6 were determined in culture supernatants by ELISA. **(F)** Cells (in triplicate) were incubated with Vi (10 μg/ml) or equivalent volume of PBS and stimulated with indicated concentrations of TNF-α. After 6 h, CXCL8 was analyzed in the supernatants by ELISA. Data is representative of 2 independent experiments. *P*-value ^***^ *p* < 0.005, NS, not significant.

The invasion of epithelial cells and induction of inflammatory responses from these cells are both initiated upon activation of small Rho family GTPases by *Salmonella* effectors such as SopE (Patel and Galán, 2006). Rac-1 plays a crucial role in enabling bacterial invasion that is tightly coupled to activation of cytoskeletal rearrangements while Cdc42 seems to be indispensable for the induction of inflammatory responses (Patel and Galán, 2006; Sun et al., 2018). As treatment with Vi inhibited invasion of epithelial cells with *Salmonella* (Figure 1C, Supplementary Figure S1), we analyzed whether incubation with this polysaccharide might alter the ability of these cells to activate Rac-1 and Cdc42 during infection with *S*. Typhimurium. The results showed that engagement of cells with Vi significantly inhibited *S*. Typhimurium-induced activation of both Rac1 and Cdc42 (Figure 2A; Supplementary Figure S2). This inhibition was associated with reduction in the formation of actin-rich foci in infected cells (Figure 2B). In untreated *S*. Typhimurium-infected Hela cells, more than 30% cells showed intense filamentous actin (F-actin) bundles as determined by binding to Phalloidin, which was reduced by more than 80% in cells infected in the presence of Vi (Figure 2B). Significantly, membrane prohibitin was found to co-localize with F-actin in cells infected with *S*. Typhimurium (Figure 2C). Consistent with suppression of Cdc42 activation, engagement of prohibitin with Vi also modulated downstream signaling intermediates that regulate inflammatory responses in infected cells. Treatment of Hela or T84 with Vi inhibited degradation of IkB-α (indicating inhibition of NF-kB pathway) and phosphorylation of JNK MAP-kinase during infection with *S*. Typhimurium (Figure 2D; Supplementary Figures S3, S4). It also delayed infection-induced serine phosphorylation of PKC-α that is known to participate in inflammatory responses during infection of epithelial cells with *Salmonella* (Figure 2D; Supplementary Figure S2; Silva et al., 2004). The inhibition brought about by Vi in the activation of these signaling intermediates resulted in significant reduction in the secretion of CXCL8 and IL-6 from infected cells (Figure 2E; Supplementary Figure S5A). The inhibitory effect of Vi on cytokine response was also seen during infection of cells with Vi negative *S*. Typhi (Supplementary Figure S5B). The reduction brought about by Vi was specific to this molecule as cells infected with *S*. Typhimurium in presence of *S*. Typhi LPS did not show reduced cytokine secretion (Figure 2E). Moreover, Vi did not inhibit CXCL8 secretion from Hela cells stimulated with TNF-α (Figure 2F). Neither Vi nor LPS induced any detectable cytokine on its own. These results demonstrated that the interaction of Vi with membrane prohibitin suppresses activation of Rho family GTPases during infection of epithelial cells with *Salmonella* resulting in reduced invasion and dampening of inflammatory responses.

**Figure 3:**
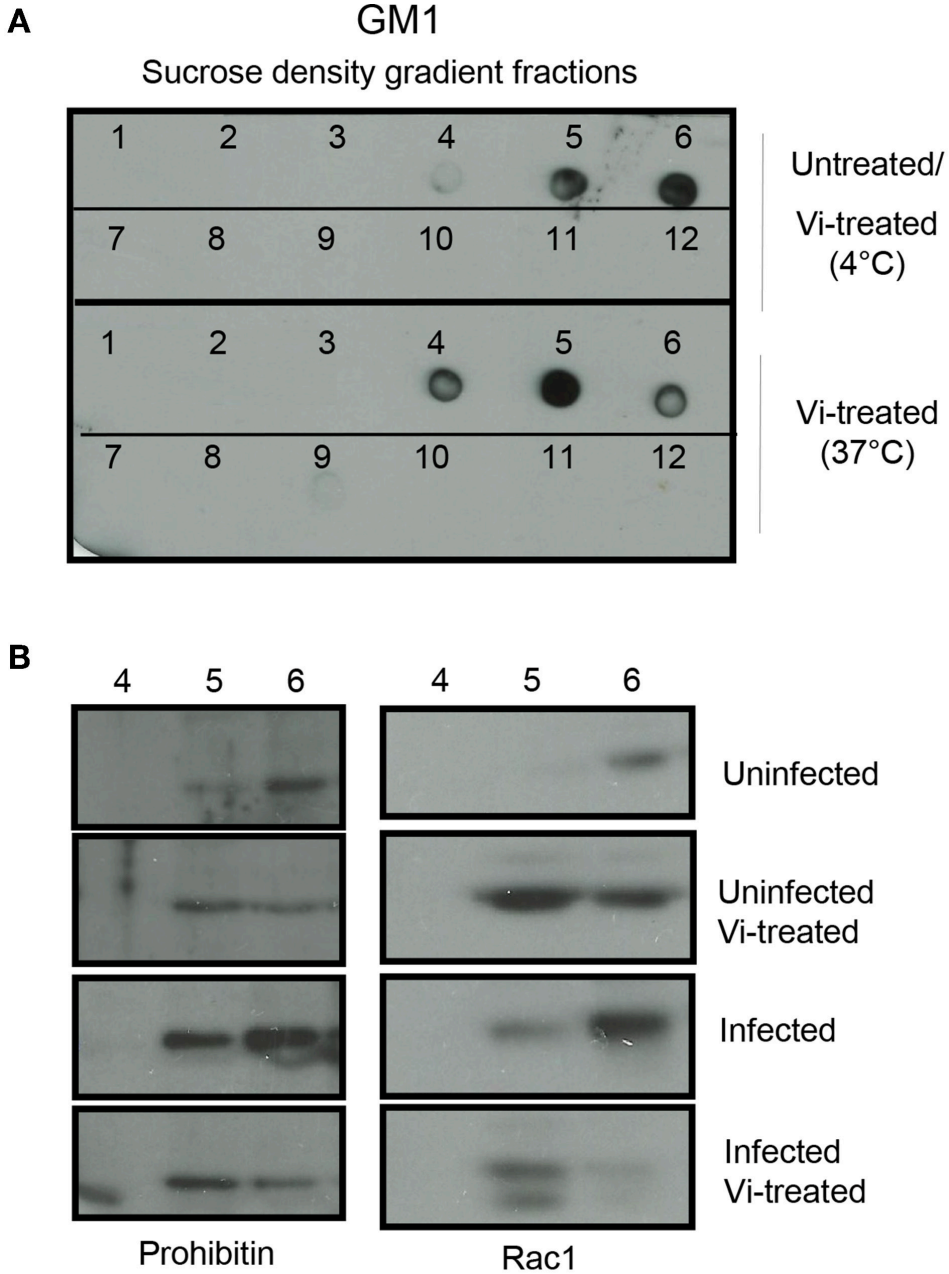
Vi brings about molecular rearrangements in the membrane raft. **(A)** Cells were left untreated or treated with Vi for 1 h at 4 or 37°C. Lipid rafts were prepared from lysates prepared from these cells by sucrose density gradient centrifugation. Fractions were absorbed on to a nitrocellulose membrane and probed with HRP-conjugated Cholera Toxin B (HRP-CTB). **(B)** Untreated or Vi-treated cells (for 1 h at 37°C) were infected with *S*. Typhimurium (100 moi for 30 min). Fractions 4, 5, and 6 were run in a 10% SDS-polyacrylamide gel, transferred to a nitrocellulose membrane and probed with antibodies to prohibitin and Rac1. Blots were developed using ECL. Data are representative of 2 independent experiments.

**Figure 4:**
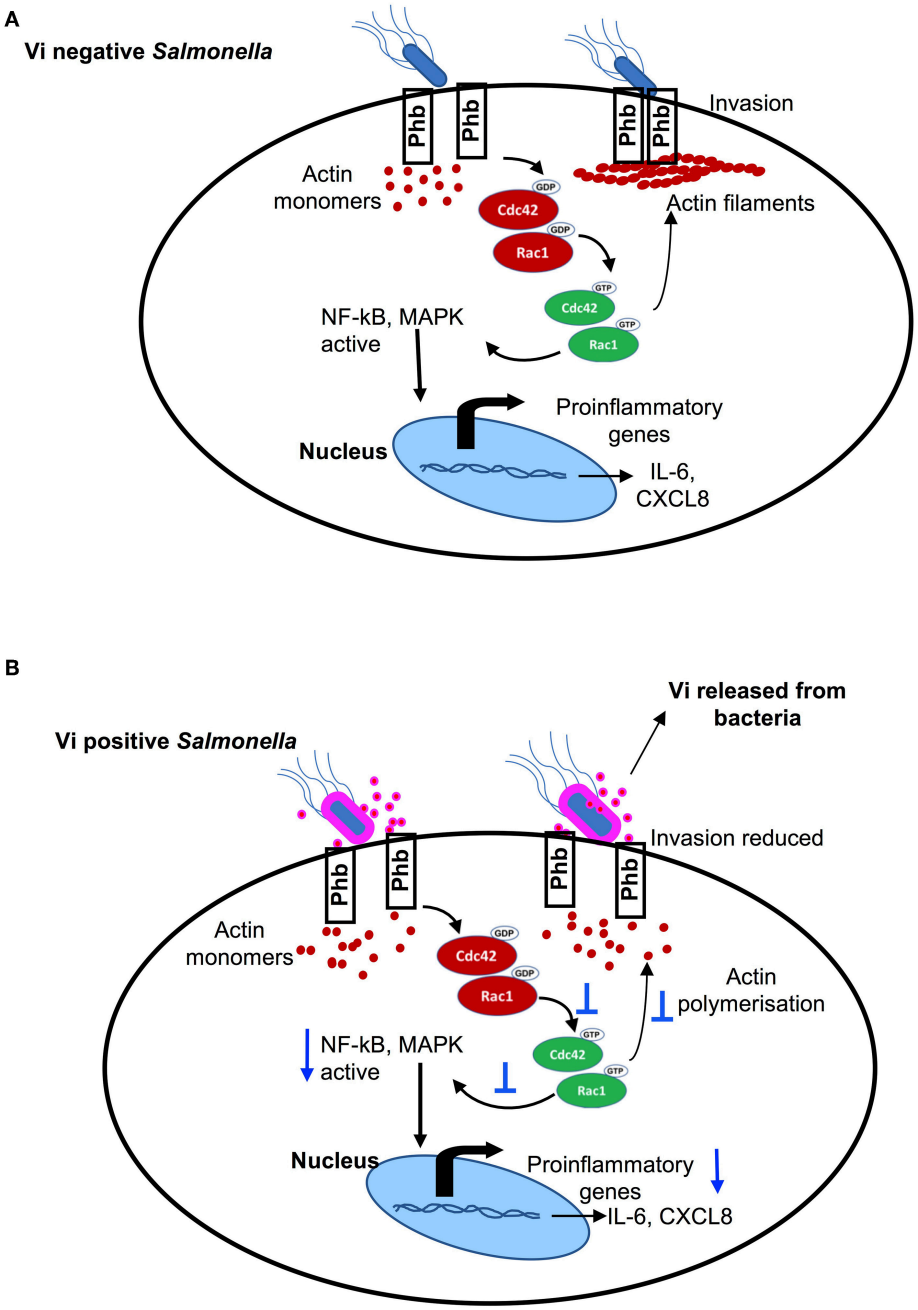
**(A)** Vi negative *Salmonella* activates small Rho family GTPases, Cdc42, and Rac-1, that bring about actin polymerisation & activation NF-kB and MAP-kinase pathways of intracellular signaling resulting in invasion and induction of inflammatory responses. **(B)** Vi present on the surface of Vi-positive *Salmonella* and released in the extracellular milieu (or Vi provided with Vi negative *Salmonella*) specifically engages membrane prohibitin that prevents activation of Cdc42 & Rac-1 resulting in reduced invasion of cells with *Salmonella* and inhibition of inflammatory responses.

The activation of GTPases is known to be regulated by trafficking of these molecules in and out of cholesterol rich raft domains (Fessler et al., 2004; Wysoczynski et al., 2005), therefore, we investigated if interaction of Vi with prohibitin, which is also a raft resident protein, might affect the localization of Rac-1 in the membrane raft. Incubation of cells with Vi at 4°C did not change the localization of bona fide raft marker, ganglioside GM1, monitored by binding to Cholera toxin B chain (CTB) (Figure 3A, this profile was identical to the one seen in untreated cells-data not shown). However, treatment of cells with Vi at 37°C consistently resulted in significant redistribution of GM1 to a lower density fraction (from fraction 6 to fractions 4 and 5 from top; Figure 3B). Prohibitin and Rac1 were found to be significantly enriched in detergent insoluble membrane fraction 6 in untreated cells (Figure 3B). Upon incubation with Vi, prohibitin and Rac1 redistributed between fractions 5 and 6 in a manner similar to that observed with GM1 (Figure 3B). Infection with *S*. Typhimurium resulted in further enrichment of prohibitin and Rac1 in fraction 6 indicating that this fraction might represent a domain that serves as the site for initiation of cellular events required for bacterial invasion and induction of inflammatory responses (Figure 3B). This enrichment was not observed in cells infected with *S*. Typhimurium in presence of Vi (Figure 3B; Supplementary Figure S6). These results suggested that engagement of membrane prohibitin with Vi brings about molecular rearrangements in the raft that prevent activation of Rho family GTPases following infection with *Salmonella* (Figure 4).

## Discussion

*S*. Typhi and *S*. Typhimurium produce different clinical outcomes in humans despite sharing a high degree of homology at the genome and the proteome level, the reasons for which are not clear (Young et al., 2002; Napolitani et al., 2018). It is believed that unlike *S*. Typhimurium, which generates a potent inflammatory response in the gut, *S*. Typhi might dampen or evade innate immune responses in the gut in order to disseminate systemically (Santos, 2014). A striking difference between these two closely related serovars is the virulence polysaccharide Vi that is present only in *S*. Typhi and forms a capsule around this pathogen. We have previously shown that Vi specifically engages membrane prohibitin in cells and reduces generation of inflammatory responses (Sharma and Qadri, 2004; Garg and Qadri, 2010). The results presented here demonstrate that targeting of prohibitin with Vi brings about changes in the membrane that prevent activation of GTPases, Rac1, and Cdc42 (Figure 2A), which play a critical role in initiating invasion and invasion-dependent inflammatory responses during infection of epithelial cells with *Salmonella* (Hardt et al., 1998). Interestingly, membrane prohibitin was found to co-localize with filamentous actin in cells infected with *S*. Typhimurium (Figure 2C). This co-localization and its perturbation with Vi resulting in reduced invasion and cytokine secretion suggests that membrane prohibitin might be a vital component of the molecular machinery that engages with *Salmonella* or *Salmonella*–derived effectors to bring about actin cytoskeletal rearrangements required for bacterial invasion and production of inflammatory responses. The exact mechanism by which Vi-prohibitin interaction prevents actin cytoskeletal reorganization is not clear at the moment. We have shown earlier that treatment of human T cells with Vi results in actin depolymerisation (Santhanam et al., 2014). It is therefore likely that redistribution of raft resident molecules including prohibitin and Rac-1 brought about by Vi in Hela cells is a result of actin depolymerisation resulting in changes in the sizes of raft domains. Treatment of T cells with actin depolymerising agent, cytochalasin D, has been shown to reduce the size of lipid raft vesicles and inhibit coalescence of rafts induced by TCR aggregation (Valensin et al., 2002). The ability of Vi to inhibit invasion through selectively bringing about changes in the lipid raft was also suggested by activation of ERK phosphorylation in Hela cells treated with Vi (unpublished data). Similar ERK activation has been previously reported in cells treated with cholesterol-depleting agent methyl β-cyclodextrin (Kabouridis et al., 2000). However, unlike methyl β-cyclodextrin, the changes brought about by Vi in the raft are not accompanied by cell death. Engagement of membrane prohibitin with Vi significantly inhibited activation of Cdc42 as well as Rac-1 during infection with *Salmonella*. These two GTPases are known to perform different functions during infection with this pathogen with Cdc42 contributing to inflammation and Rac-1 regulating pathogen invasion (Patel and Galán, 2006; Sun et al., 2018). It is therefore tempting to speculate that Vi-mediated changes in the membrane raft may alter the “host signal” whose sensing by *Salmonella* is needed to trigger release of invasion and inflammation-promoting molecules from the pathogen (Hardt et al., 1998). In this context, it is already known that one of the *Salmonella* effectors involved in bringing about invasion and cell death, SipB, binds cholesterol with very high affinity (Hayward et al., 2005). Irrespective of the exact mechanism by which Vi brings about changes in actin cytoskeleton, details of which are currently being worked out in our laboratory, the results shown here reveal a previously unappreciated mechanism by which this virulence polysaccharide can reduce invasion and induction of inflammatory responses during infection of epithelial cells with *S*. Typhi. Wilson et al. (2008) and Winter et al. (2008) have shown previously that Vi can downregulate inflammatory responses from cells by regulating release and/or expression of TLR ligands from *S*. Typhi. Our results suggest that in addition to modulating release of bacterial effectors, Vi can directly target membrane prohibitin in host cells and reduce invasion and invasion-dependent inflammatory responses *via* inhibition of activation of Rho family GTPases. Through this interaction, Vi released from *S*. Typhi might in fact prime cells for reduced invasion and consequently reduced inflammatory response. This modulatory pathway might also be relevant to anti-phagocytic activity of Vi in macrophages (Looney and Steigbigel, 1986; Wilson et al., 2011). Thus, our findings reveal an efficient strategy that might be employed by *S*. Typhi to limit its invasion and thereby reduce its sensing by the host. Such a strategy would dampen innate immune responses and promote establishment of systemic infection. Together with our previous studies, these findings also suggest that membrane prohibitin might have a broader regulatory role in controlling actin cytoskeletal rearrangements critically required for a variety of cellular processes including receptor signaling, phagocytosis and induction of inflammatory responses.

## Experimental Procedures

### Cells, Antibodies, and Other Reagents

Human cervical epithelial cell line, Hela, was obtained from the American Type Culture Collection (ATCC). Cells were maintained in DMEM supplemented with 10% FCS (DMEM-10) at 37°C in a 5% CO_2_ incubator. All antibodies used in this study were purchased from Cell Signaling Technology or Millipore. Polyclonal rabbit antibody against prohibitin was previously generated in the laboratory as described by Coates et al., (Coates et al., 1997). Vi was obtained from Bharat Biotech Limited, India. It was dialyzed against PBS before using in cellular experiments. Monoclonal antibody against Vi has been described previously (Qadri et al., 1990). PBD [Cdc42/Rac1 (p21)-binding domain]-GST expression vector was kindly provided by Dr. Sourav Banerjee, National Brain Research Center, Manesar, India. It was expressed in *E. coli* and purified by affinity chromatography using glutathione agarose beads as described by Patel and Galán (2006). CXCL8 and IL-6 detection kits were obtained from BD Biosciences.

### Bacterial Strains and Culture

Vi^+^ and Vi^−^ clinical isolates of *S*. Typhi were kindly provided by Prof. Geeta Mehta, Department of Microbiology, Lady Hardinge Medical College, New Delhi, India. *S*. Typhimurium SL1344 was provided by Prof. Emmanuelle Charpentier, Department of Microbiology and Genetics, University of Vienna, Austria and GFP-expressing *S*. Typhimurium by Amitabha Mukhopadhyay, National Institute of Immunology, New Delhi. *S*. Typhimurium was grown in Luria Bertani (LB) broth supplemented with streptomycin (100 μg/ml for SL1344) or ampicillin (100 μg/ml for GFP^+^ *S*. Typhimurium) at 37°C with overnight shaking (220 rpm).

### Analysis of Vi-Release During Infection of Cells With *S. T*yphi

0.3 × 10^6^ Hela cells seeded in a 24-well tissue culture plate were infected with Vi positive and Vi negative *S*. Typhi (10 MOI) for 1 h. Extracellular medium was collected and filtered through 0.22μ membrane to get rid of bacteria and incubated with freshly plated Hela cells. After washing, cells were incubated with anti-Vi MoAb followed by PE-labeled anti-mouse IgG antibody. Cells were analyzed by flow cytometry. The binding of purified Vi to Hela cells was determined in a similar fashion. All the experiments with *Salmonella* were carried out in a BSL-2 facility as per the guidelines provided by the Institutional Biosafety Committee.

### Infection of Cells With *Salmonella*

0.3 × 10^6^ Hela cells were seeded in a 24-well tissue culture plate 12–16 h before infection. Cells were infected with GFP^+^ *S*. Typhimurium (50 MOI) in the absence or presence of indicated concentrations of Vi, washed to remove unbound bacteria and incubated for another 30 min in presence of gentamycin (100 μg/ml). Cells were fixed with 0.2% paraformaldehyde and analyzed by flow cytometry for presence of intracellular bacteria.

For cytokine analysis, cells were incubated for 30 min with different concentrations of Vi or LPS before infecting with bacteria at different MOIs. Thirty minutes later, cells were washed and incubated in RPMI-1640 containing gentamycin for another 6 h. CXCL8 and IL-6 were determined in the supernatants by ELISA. Cells were also stimulated with TNF-α in the absence or presence of Vi and CXCL8 was analyzed after 6 h. Unless mentioned otherwise, all cell stimulations were carried out under serum-free conditions.

For adhesion, cells were incubated with bacteria at 4°C, washed to remove unbound bacteria and cell lysates were plated on SS-Agar to determine CFU.

### Immunoprecipitation

Immunoprecipitation with Vi was carried out as described previously (Sharma and Qadri, 2004). Briefly, Hela cells were washed with serum-free RPMI-1640 and incubated at 4°C with Vi (1 μg/ml/5 × 10^6^ cells) or equivalent volume of PBS. Cells were washed three times with PBS and lysed in 1 ml ice-cold lysis buffer (TKM-Triton X-100 1%, containing a cocktail of protease inhibitors) for 30 min on ice. Cell lysates were cleared at 10,000 × g for 20 min, filtered through a 0.45 μm filter and loaded onto Protein G-Sepharose beads preloaded with anti-Vi MoAb. After 4 h, beads were washed with TKM lysis buffer (50 mM Tris-HCl, pH 7.6, 200 mM NaCl, 10 mM MgCl_2_, 1 mM PMSF, 5% (v/v) Glycerol, and 1% Triton X-100), boiled with Laemmli sample buffer (non-reducing) and run in a 12% SDS-Polyacrylamide gel. The proteins were transferred to a NC membrane, blotted with rabbit polyclonal anti-prohibitin antibody followed by HRP-labeled anti-rabbit IgG antibody. The blot was developed using ECL.

### Determination of Rho-GTPase Activation

Hela cells were seeded on two 10-cm petri dishes 12–16 h prior to start of the experiment. Cells were treated with Vi (10 μg/ml) or PBS for 30 min, and infected with *S*. Typhimurium at an MOI of 100 for 30 min. Cells were washed twice with cold PBS and lysed in TKM lysis buffer containing a cocktail of protease inhibitors. Cell lysates were centrifuged at 12,000 × g for 10 min at 4°C and incubated for 1 h at 4°C with PBD-GST pre-loaded onto glutathione agarose beads. Beads were washed twice, boiled with Laemmli sample buffer and proteins resolved by SDS-PAGE were transferred to nitrocellulose membrane. The membrane was probed with anti-Cdc42 or Rac1 antibodies followed by HRP-labeled rabbit anti-mouse Ig antibody. The blot was developed using ECL and band intensities of activated Cdc42 and Rac-1 were determined using ImageJ program.

### Analysis of Actin Polymerisation

Cells were incubated with PBS or Vi (5 μg/ml) at 37°C followed by infection with *S*. Typhimurium (100 MOI) at 37°C for 30 min. Cells were washed, fixed with 4% paraformaldehyde for 20 min at room temperature and stained with Alexafluor 488-labeled Phalloidin to visualize filamentous actin (F-actin). Several fields were visually scanned under the microscope to determine approximate number of F-actin bundles in infected cells in the absence and presence of Vi. Images were acquired in a fluorescent microscope (Nikon TE-2000) and imported into Adobe-Photoshop.

To analyze co-localization of F-actin and membrane prohibitin, cells were left uninfected or infected with *S*. Typhimurium for 30 min and stained for F-actin with Alexafluor-488-labeled Phalloidin (green staining). Subsequently, cells were incubated with Vi at 4°C (for staining membrane prohibitin) followed by anti-Vi antibody and Alexafluor 594-labeled anti-mouse Ig Ab (red staining).

### Analysis of Intracellular Signaling

0.3 × 10^6^ cells were incubated with Vi (10 μg/ml) for 30 min and infected with *S*. Typhimurium for different time points. Cold PBS was added to stop activation. Cells were detached using EDTA and after washing, lysed in TKM buffer containing 1% Triton X-100 and a cocktail of protease inhibitors. Lysates were boiled with a Laemmli sample buffer and electrophoresed in 10% SDS-polyacrylamide gel followed by transfer to a nitrocellulose (NC) membrane. The NC membrane was probed with commercially available antibodies against p-JNK, IkB-α, phospho-PKC-α, and GAPDH followed by HRP-labeled anti-rabbit Ig antibodies. The blots were developed using ECL and band intensities of activated Cdc42 and Rac-1 were determined using ImageJ program.

### Lipid Raft Preparation

Lipid rafts from infected and uninfected Hela cells were prepared as described by Xavier et al. (1998). Briefly, 3 × 10^7^ cells were washed with PBS, lysed in 500 μl TKM buffer supplemented with 1% Triton X-100 containing protease inhibitor cocktail for 30 min on ice. The lysate was homogenized with 10 strokes of a Dounce hand homogeniser and centrifuged at 500 × g for 10 min. The supernatant was mixed with an equal volume of 85% sucrose (w/v) prepared in TKM buffer and placed at the bottom of a centrifuge tube (SW55Ti Thermo centrifuge). The sample was overlaid with 3 ml of 35% sucrose and 2 ml of 5% sucrose prepared in TKM, and centrifuged at 200,000 × g for 14–16 h at 4°C. Twelve fractions of 400 μL each were collected.

The localization of raft marker GM1 in different fractions was determined by reactivity with Cholera Toxin B (CTB) chain in a dot-blot assay. Briefly, 3 μl of each fraction was spotted on a nitrocellulose membrane and allowed to dry at room temperature. The membrane was blocked with 1% bovine serum albumin for 1 h and incubated after washing with HRP-labeled CTB for 1 h at 37°C. The membrane was washed extensively with PBS-Tween-20 (0.5%) and the spots were visualized using ECL as described above for western blotting.

The fractions enriched in GM1 were run in an SDS-polyacrylamide gel, transferred to nitrocellulose membrane and probed with antibodies against prohibitin and Rac-1. The blot was developed using ECL.

### Statistical Analysis

Student's *t*-test with a two tailed distribution with type 3 test for unequal variances was used to calculate *p*-values. A *p*-value of <0.05 was considered statistically significant. Data are expressed as mean ± SD. Error bars represent standard deviation (SD).

## Author Contributions

AQ conceived and supervised the study. FP, JY, and AQ designed experiments. FP and JY performed experiments and prepared data for publication. FP, JY, and AQ analyzed the data and wrote the manuscript. All authors approved the manuscript.

### Conflict of Interest Statement

The authors declare that the research was conducted in the absence of any commercial or financial relationships that could be construed as a potential conflict of interest.
