# Supplementary material for: The Virulence Polysaccharide of Salmonella Typhi Suppresses Activation of Rho Family GTPases to Limit Inflammatory Responses From Epithelial Cells
Source: Front Cell Infect Microbiol. 2019 May 8;9:141. doi: 10.3389/fcimb.2019.00141 (PMC6517557; doi:10.3389/fcimb.2019.00141)
Supplement: Supplementary file 1 [file Presentation_1.ppt]

## Slide 1
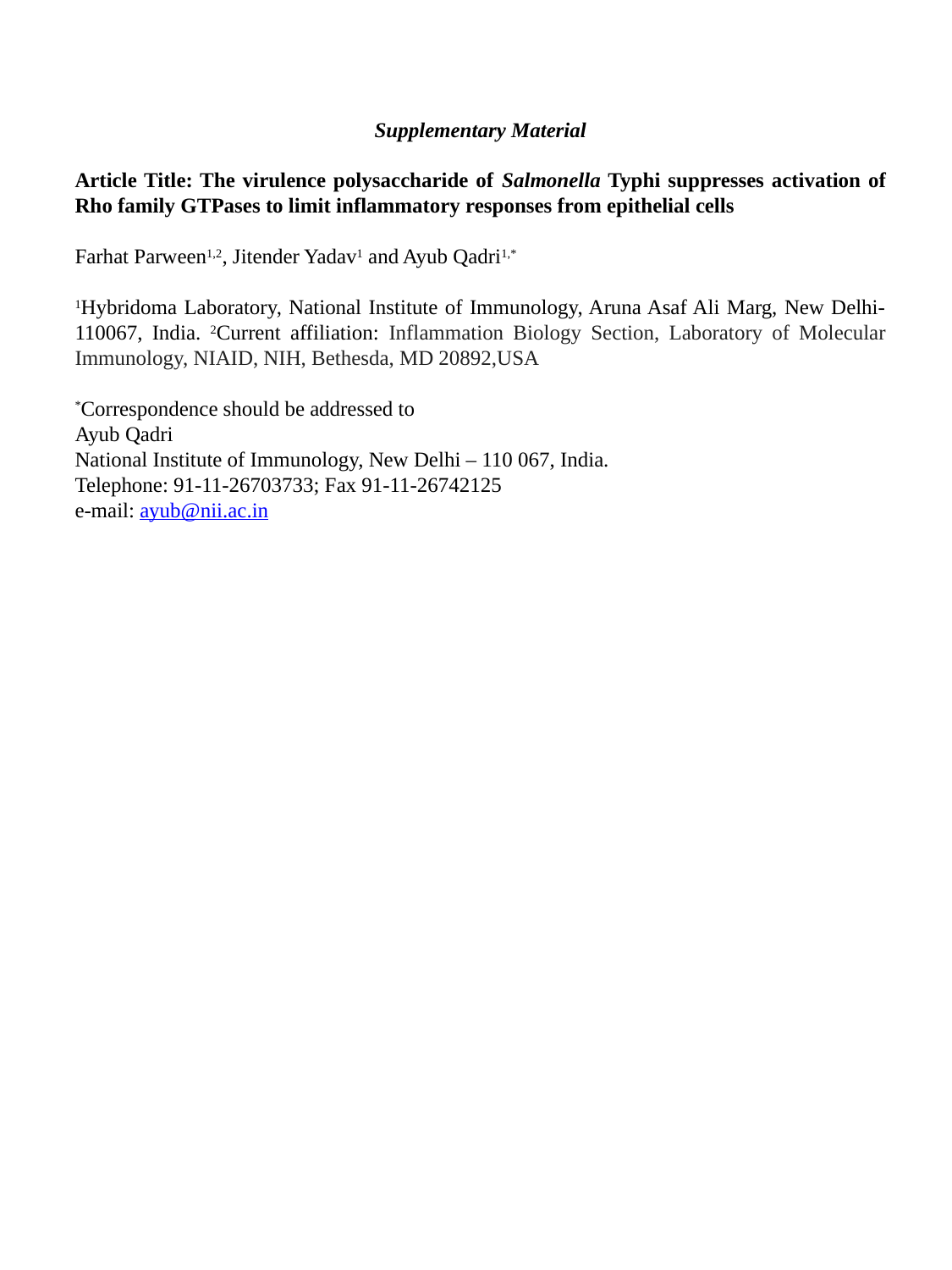

Supplementary Material
Article Title: The virulence polysaccharide of Salmonella Typhi suppresses activation of Rho family GTPases to limit inflammatory responses from epithelial cells
Farhat Parween1,2, Jitender Yadav1 and Ayub Qadri1,*
1Hybridoma Laboratory, National Institute of Immunology, Aruna Asaf Ali Marg, New Delhi-110067, India. 2Current affiliation: Inflammation Biology Section, Laboratory of Molecular Immunology, NIAID, NIH, Bethesda, MD 20892,USA
*Correspondence should be addressed to
Ayub Qadri
National Institute of Immunology, New Delhi – 110 067, India.
Telephone: 91-11-26703733; Fax 91-11-26742125
e-mail: ayub@nii.ac.in

## Slide 2
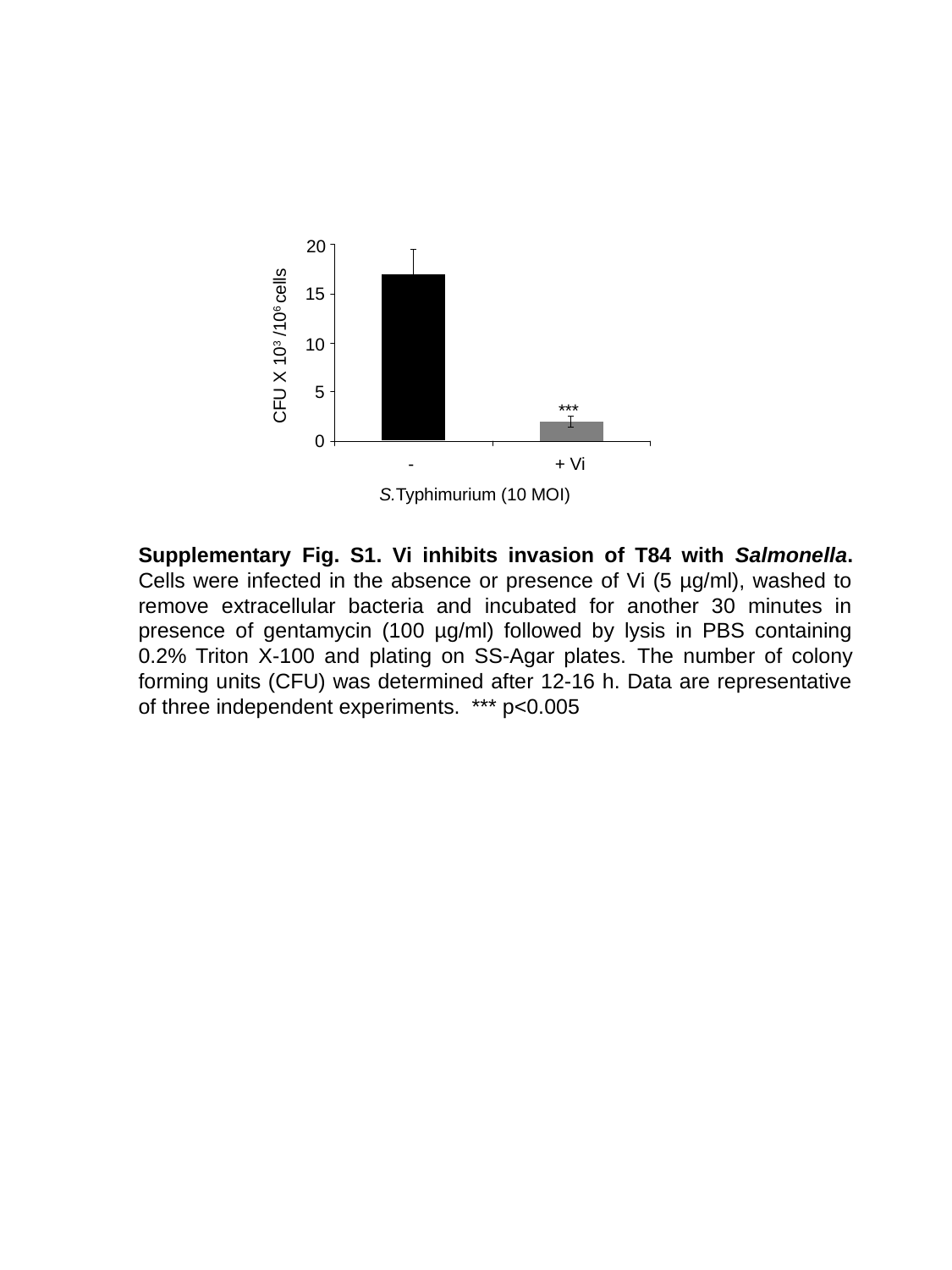

20
15
10
CFU X 103 /106 cells
5
***
0
 - + Vi
S.Typhimurium (10 MOI)
Supplementary Fig. S1. Vi inhibits invasion of T84 with Salmonella. Cells were infected in the absence or presence of Vi (5 µg/ml), washed to remove extracellular bacteria and incubated for another 30 minutes in presence of gentamycin (100 µg/ml) followed by lysis in PBS containing 0.2% Triton X-100 and plating on SS-Agar plates. The number of colony forming units (CFU) was determined after 12-16 h. Data are representative of three independent experiments. *** p˂0.005

## Slide 3
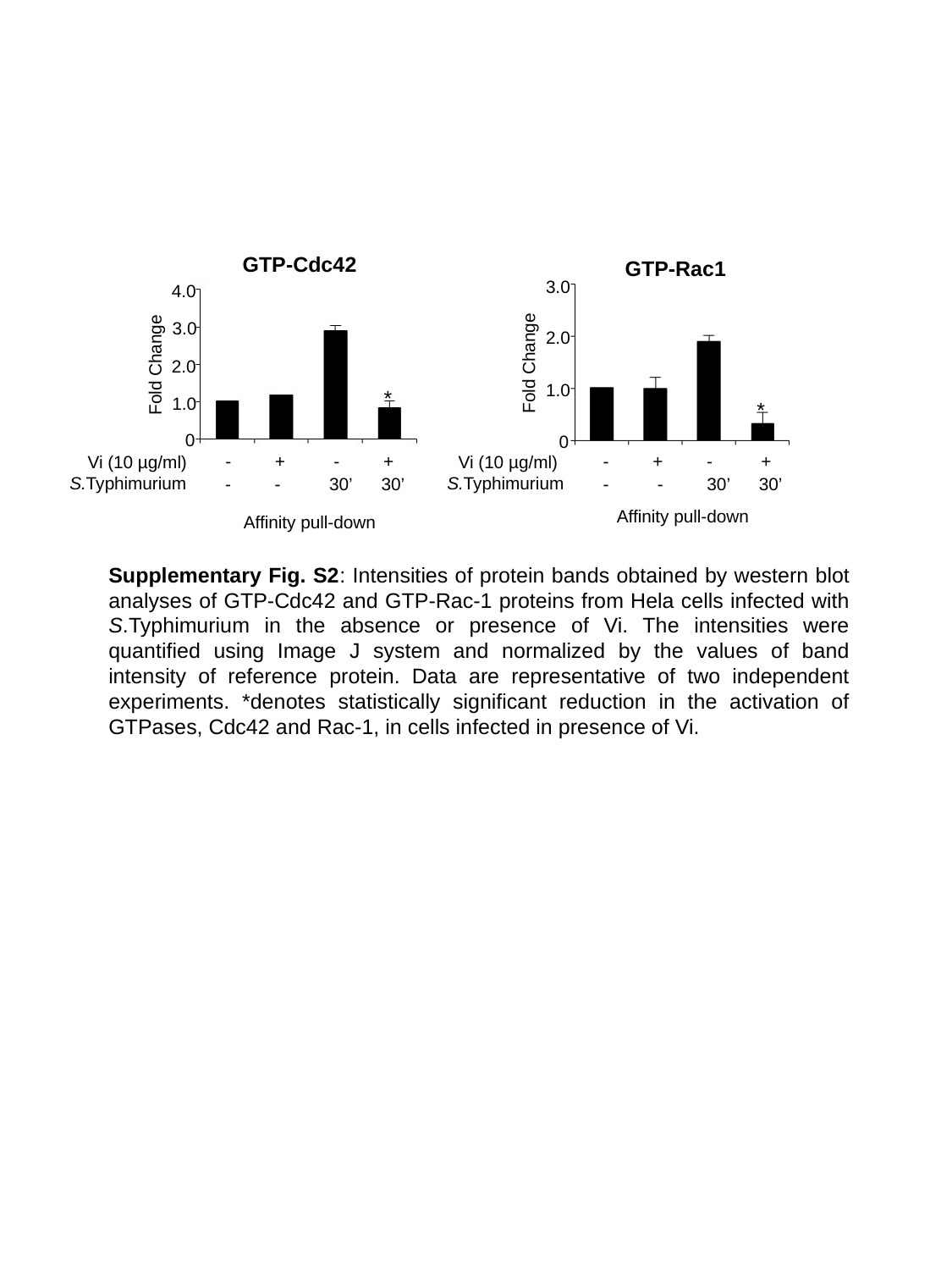

GTP-Cdc42
GTP-Rac1
3.0
4.0
3.0
2.0
Fold Change
Fold Change
2.0
1.0
*
1.0
*
0
0
 - + - +
 - + - +
 Vi (10 µg/ml)
 Vi (10 µg/ml)
S.Typhimurium
S.Typhimurium
 - - 30’ 30’
 - - 30’ 30’
Affinity pull-down
Affinity pull-down
Supplementary Fig. S2: Intensities of protein bands obtained by western blot analyses of GTP-Cdc42 and GTP-Rac-1 proteins from Hela cells infected with S.Typhimurium in the absence or presence of Vi. The intensities were quantified using Image J system and normalized by the values of band intensity of reference protein. Data are representative of two independent experiments. *denotes statistically significant reduction in the activation of GTPases, Cdc42 and Rac-1, in cells infected in presence of Vi.

## Slide 4
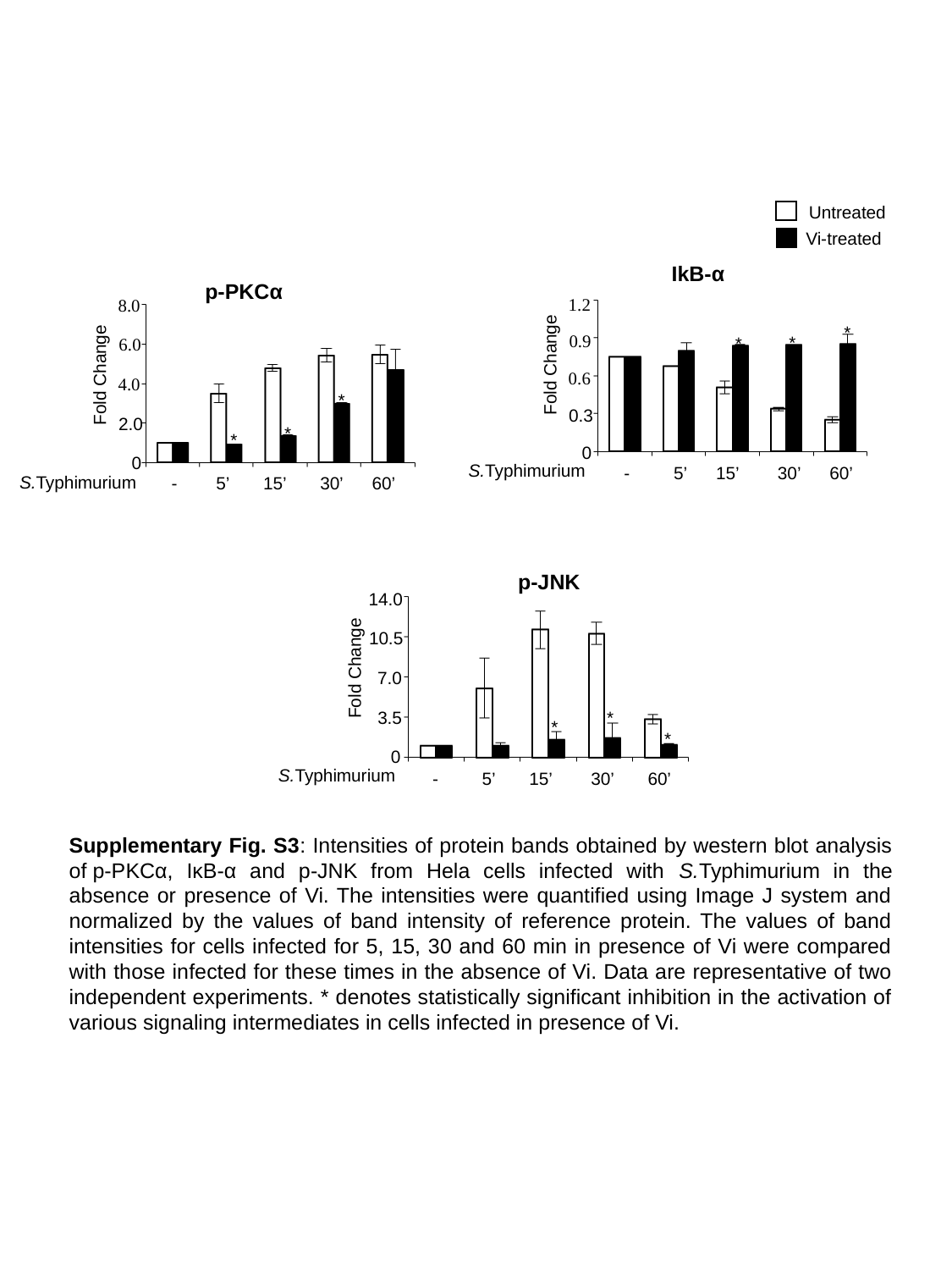

Untreated
Vi-treated
IkB-α
p-PKCα
1.2
8.0
*
0.9
*
*
6.0
Fold Change
Fold Change
0.6
4.0
*
0.3
2.0
*
*
0
0
S.Typhimurium
 - 5’ 15’ 30’ 60’
S.Typhimurium
 - 5’ 15’ 30’ 60’
p-JNK
14.0
10.5
Fold Change
7.0
*
3.5
*
*
0
S.Typhimurium
 - 5’ 15’ 30’ 60’
Supplementary Fig. S3: Intensities of protein bands obtained by western blot analysis of p-PKCα, IκB-α and p-JNK from Hela cells infected with S.Typhimurium in the absence or presence of Vi. The intensities were quantified using Image J system and normalized by the values of band intensity of reference protein. The values of band intensities for cells infected for 5, 15, 30 and 60 min in presence of Vi were compared with those infected for these times in the absence of Vi. Data are representative of two independent experiments. * denotes statistically significant inhibition in the activation of various signaling intermediates in cells infected in presence of Vi.

## Slide 5
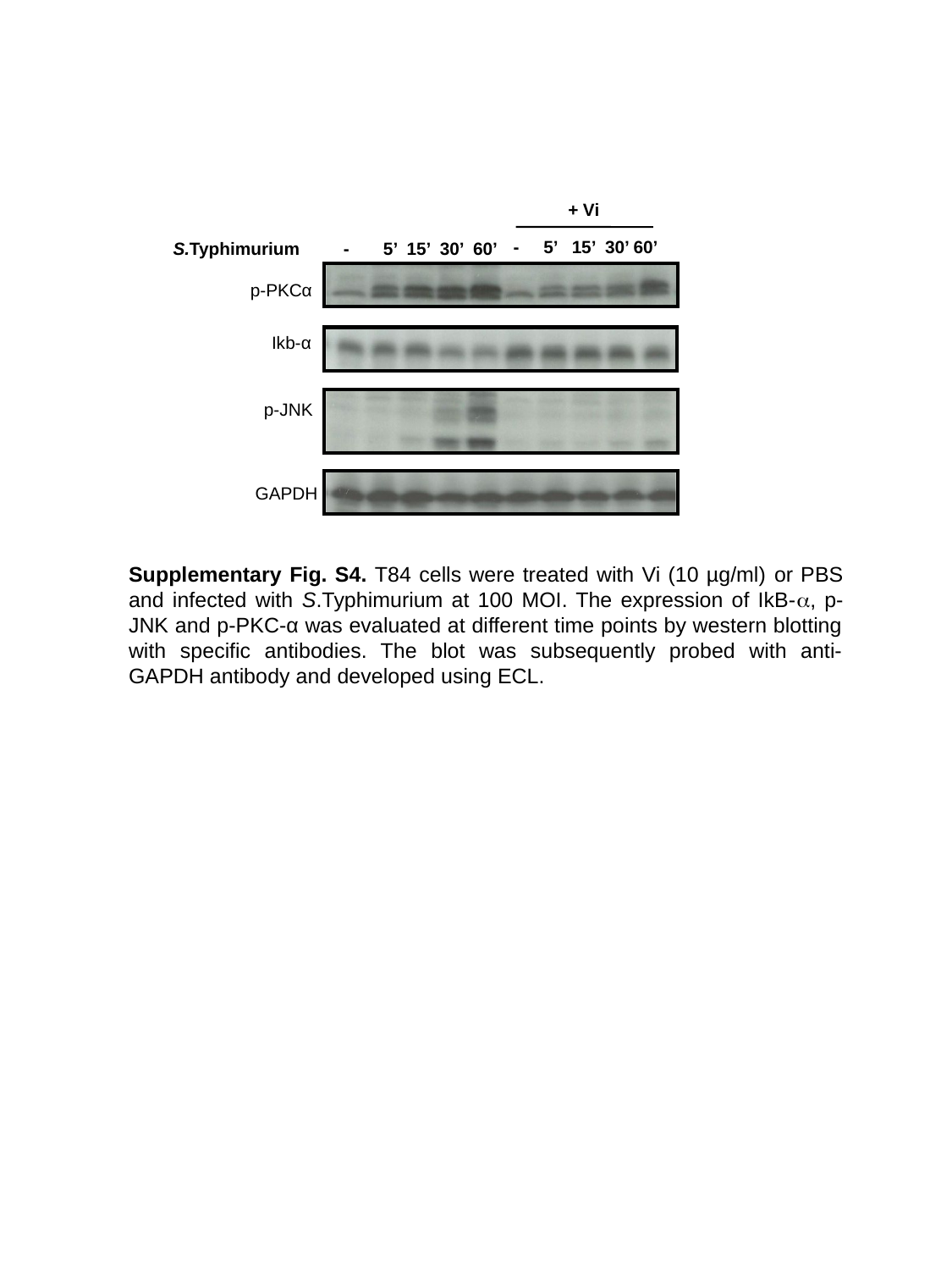

+ Vi
 - 5’ 15’ 30’ 60’
 - 5’ 15’ 30’ 60’
 S.Typhimurium
p-PKCα
Ikb-α
p-JNK
GAPDH
Supplementary Fig. S4. T84 cells were treated with Vi (10 µg/ml) or PBS and infected with S.Typhimurium at 100 MOI. The expression of IkB-α, p-JNK and p-PKC-α was evaluated at different time points by western blotting with specific antibodies. The blot was subsequently probed with anti-GAPDH antibody and developed using ECL.

## Slide 6
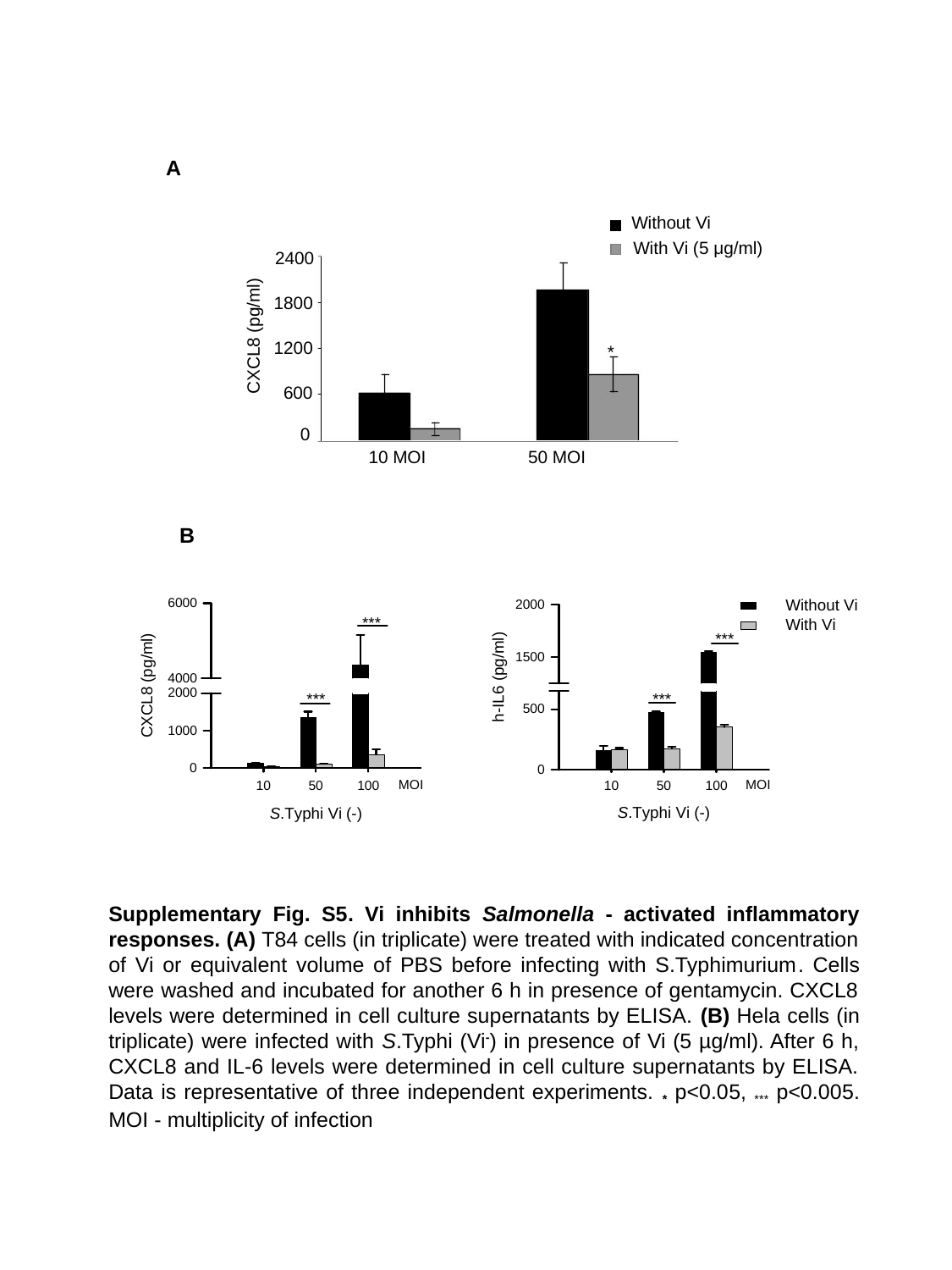

A
Without Vi
With Vi (5 μg/ml)
2400
1800
CXCL8 (pg/ml)
1200
*
600
0
10 MOI 50 MOI
B
Supplementary Fig. S5. Vi inhibits Salmonella - activated inflammatory responses. (A) T84 cells (in triplicate) were treated with indicated concentration of Vi or equivalent volume of PBS before infecting with S.Typhimurium. Cells were washed and incubated for another 6 h in presence of gentamycin. CXCL8 levels were determined in cell culture supernatants by ELISA. (B) Hela cells (in triplicate) were infected with S.Typhi (Vi-) in presence of Vi (5 µg/ml). After 6 h, CXCL8 and IL-6 levels were determined in cell culture supernatants by ELISA. Data is representative of three independent experiments. * p<0.05, *** p<0.005. MOI - multiplicity of infection

## Slide 7
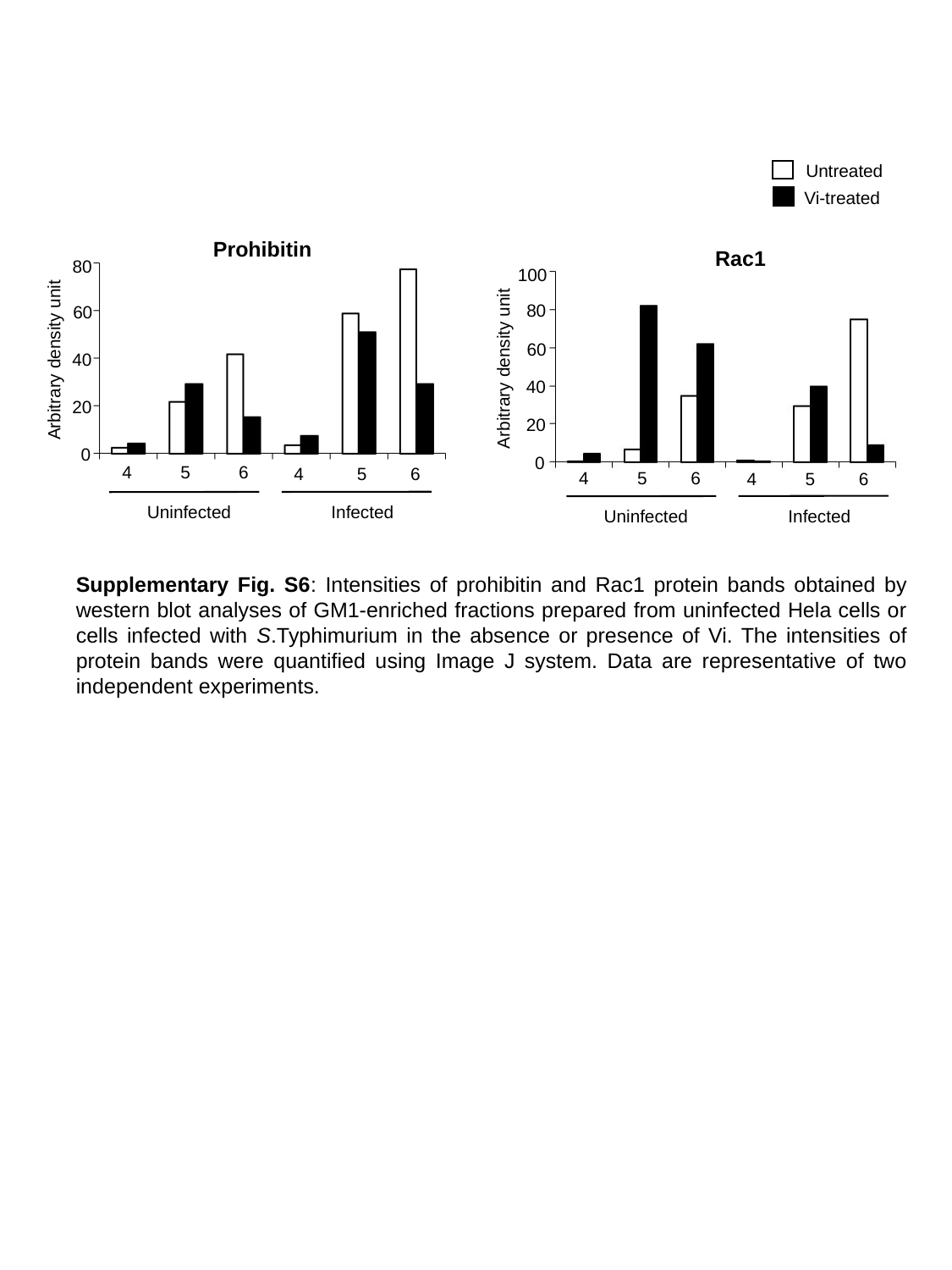

Untreated
Vi-treated
Prohibitin
Rac1
80
100
80
60
60
40
Arbitrary density unit
Arbitrary density unit
40
20
20
0
0
4 5 6
4 5 6
4 5 6
4 5 6
Uninfected
Infected
Uninfected
Infected
Supplementary Fig. S6: Intensities of prohibitin and Rac1 protein bands obtained by western blot analyses of GM1-enriched fractions prepared from uninfected Hela cells or cells infected with S.Typhimurium in the absence or presence of Vi. The intensities of protein bands were quantified using Image J system. Data are representative of two independent experiments.
